# Supplementary material for: Patterns of Intron Gain and Loss in Fungi
Source: PLoS Biol. 2004 Nov 30;2(12):e422. doi: 10.1371/journal.pbio.0020422 (PMC532390; doi:10.1371/journal.pbio.0020422)
Supplement: Table S1 — Also available at http://genes.mit.edu/NielsenEtAl/. (4.3 MB ZIP). [file pbio.0020422.st001.zip › NielsenEtAl/html/1257.html]

AN1339.1.NCU03947.1.MG07255.1.FG10272.1


```
 CLUSTAL W (1.82) Multiple Sequence Alignments - Introns Inserted


Sequence 1: MG07255.1	791 aa
Sequence 2: FG10272.1	804 aa
Sequence 3: NCU03947.1	806 aa
Sequence 4: AN1339.1	821 aa
Alignment Length: 834 aa
Number Identitical Residues: 541 aa
Alignment Score (without introns) 23572


MG07255.1 	-------~--------~---~--MYK~RDVFR1FPDPFAVATINGEQTKTTSVSKRTLNP
NCU03947.1	MTSSRD-~------SG~LPA2QPNLR0RDVFR1FPDPFAVATINGEQTKTTQVSKRTLNP
FG10272.1 	MTFNIDI~I-----QV~IAA~DGLYK~RDVFR1FPDPFAVATINGEQTKTTTVSKRTLNP
AN1339.1  	MGSNLPA2QPNLRVTI1IAA~DGLYK~RDVFR1LPDPFAVATVGGEQTHTTSVIKKTLNP
          	   .     ..       .: .   : ***** :********:.****:** * *:****

MG07255.1 	YWNESFDF2RVNEDSILAVQVFDQKKFKKKDQGFLGVINIRIGDII-ELAADADD1Q~ML
NCU03947.1	YWNEHFDF2KVNEDSILAVQVFDQKKFKKKDQGFLGVINVRIGDVI-ELAPDAED1Q~ML
FG10272.1 	YWNESFDF2RTNEDGILAVQVFDQKKFKKKDQGFLGVINIRVGDVIPELSADADD1Q~ML
AN1339.1  	YWNEMFDL2RVNEDSILAIQIFDQKKFKKKDQGFLGVINVRIGDVI-DLQMGG-D~E1ML
          	**** **: :.***.***:*:******************:*:**:* :*  .. * : **

MG07255.1 	TRDLKKSTDNLVVHGKLIVNLSTNLTTPARGQQQLAIPHRPAVGTPQPSSASSLAAGTVD
NCU03947.1	TRDLKKSTDNLVVHGKLIINLSTNLTATMSRLGPPPSSSRPSLLTP---QSSVISNDRAN
FG10272.1 	TRDLKKSTDNLVVHGKLIINLSCNLSAPARGGQ--TSTARPSLGTG-PSNASNLSAPPPE
AN1339.1  	TRDLKKSNDNLVVHGKLIINLSTNLSTPNPNQANGLHRTQLGASTSSGLVPQVAPTPSVP
          	*******.**********:*** **::.           : .  * .   ..  .     

MG07255.1 	NRPNSAMGGSNGSTHSPQ-PAHRPV--SMNATPTVPQNG--ASTQN----------RNSS
NCU03947.1	ERPSSAMSGPNGTANNMT-LASRPASLAVSSSSTAPTPGTNGTAPTNPSTLVPAQARHHS
FG10272.1 	NRPGSSMSGPNGAGGSQVNLAHRPS--SINSVGGASATGPNASGPT----------RQAN
AN1339.1  	QAGPSSVDQSAAASSASLNPQRVPS-ATRPTSQIAPPNGAPPIANG--------QGVPRP
          	:   *::. . .:     .    *   :  :   ..  *.              ..    

MG07255.1 	AMSPFEDAQGRLPAGWERREDNLGRTYYVDHNTRTTSWNRPTAAGGAAESRNAQEAATQV
NCU03947.1	TLSPFEDSMGRLPAGWERREDHLGRTYYVDHNSRTTSWNRPTGTG-AAENR-TAEANTQV
FG10272.1 	QLSPFEDAQGRLPAGWERREDNLGRTYYVDHNTRTTSWNRPTATG-AQEQRNDREAATQV
AN1339.1  	NLSSFEDNQGRLPAGWERREDNLGRTYYVDHNTRTTTWNRPSANYNEQTQRTQREANMQL
          	 :*.***  ************:**********:***:****:.  .   .*.  **  *:

MG07255.1 	ERQRHQNRTLPEDRTGANSPTLQQQQQQQQQ-----AAAQAANNQTMMHTGATSPGTGEL
NCU03947.1	ERQRHQNRTLPEDRTGANSPTLQQQ-----------QAAATANAATMMHTGATTPGTGEL
FG10272.1 	ERQRHQNRTLPEERTGSNSPTMHAQQPQP-------AASPATNGGAVMHTGATSPGTGEL
AN1339.1  	ERRAHQNRMLPEDRTGASSPNLSETQPQAQTPPAGGSGASNSNVVSMMATGATTAGTGEL
          	**: **** ***:***:.**.:   . . . ..:.. .:  :*  ::* ****:.*****

MG07255.1 	PPGWEQRFTPEGRPYFVDHNTRTTTWVDPRRQQYIRMYGGQNNANG--TIQQQPVSQLGP
NCU03947.1	PAGWEQRFTPEGRPYFVDHNTRTTTWVDPRRQQYIRMYGGQNNTNG--TIQQQPVSQLGP
FG10272.1 	PPGWEQRWTPEGRPYFVDHNTRTTTWVDPRRQQYIRMYGGQNNANG--QIQQQPVSQLGP
AN1339.1  	PPGWEQRTTPEGRPYFVDHNTRTTTWVDPRRQQYIRMYG-QNASGGNTTIQQQPVSQLGP
          	*.***** ******************************* ** :.*.: ***********

MG07255.1 	LPSGWEMRLTNTARVYFVDHNTKTTTWDDPRLPSSLDQNVPQYKRDFRRKLIYFRSQPAM
NCU03947.1	LPSGWEMRLTNTARVYFVDHNTKTTTWDDPRLPSSLDQNVPQYKRDFRRKLIYFRSQPAM
FG10272.1 	LPSGWEMRLTNTARVYFVDHNTKTTTWDDPRLPSSLDQNVPQYKRDFRRKLIYFRSQPAM
AN1339.1  	LPSGWEMRLTNTARVYFVDHNTKTTTWDDPRLPSSLDQGVPQYKRDFRRKLIYFRSQPAL
          	**************************************.********************:

MG07255.1 	RILQGQCHIKVRRSHIFEDSFAEISRQSATDLKKRLMIKFDGEDGLDYGGLSR~EFFFLL
NCU03947.1	RIMSGQCHIKVRRSHIFEDSFAEISRQSATDLKKRLMIKFDGEDGLDYGGLSR~EFFFLL
FG10272.1 	RILSGQCHIKVRRSHIFEDSFAEITRQSATDLKKRLMIKFDGEDGLDYGGLSR~EFFFLL
AN1339.1  	RIMSGQCHVKVRRNNIFEDSYAEIMRQSASDLKKRLMIKFDGEDGLDYGGLSR2EFFFLL
          	**:.****:****.:*****:*** ****:*********************** ******

MG07255.1 	SHEMFNPFYCLFEYSAHDNYTLQINPHSGINPEHLNYFKFIGRVVGLAIFHRRFLDAFFI
NCU03947.1	SHEMFNPFYCLFEYSAHDNYTLQINPHSGINPEHLNYFKFIGRVVGLAIFHRRFLDAFFI
FG10272.1 	SHEMFNPFYCLFEYSAHDNYTLQINPHSGINPEHLNYFKFIGRVVGLAIFHRRFLDAFFI
AN1339.1  	SHEMFNPFYCLFEYSAHDNYTLQINPHSGVNPEHLNYFKFIGRVVGLAIFHRRFLDSFFI
          	*****************************:**************************:***

MG07255.1 	GALYKMVLGKSVALPDMEGVDADFHRSLQWML~DNDISGGILEQTFSTEDERFGVITVED
NCU03947.1	GALYKMVLGKAVSLADMEGVDADFHRSLQWML~DNDIT-DVLDATFSTEDERFGVITEED
FG10272.1 	GALYKMMLGKAVALADMEGVDADFHRSLQWML~DNDISGGILEQTFSTEDERFGVLTTED
AN1339.1  	GAFYKMMLRKKVSLQDMEGVDEDLHRNLTWTL2ENDIE-GIIDLTFTVDDEKFGERRTIE
          	**:***:* * *:* ****** *:**.* * * :***  .::: **:.:**:**     :

MG07255.1 	LIPNGRNIDVTNENKKEYVD~LMVKWRIEKRIAEQFEAFKTGFHELIPQDLINVFDEREL
NCU03947.1	LIPNGRNIAVTNENKKEYVE~LMVKWRIEKRIEQQFRAFKDGFHELIPQDLINVFDEREL
FG10272.1 	LIPGGRDIEVTNENKKEYVD~LMVKWRIEKRIAEQFQAFKEGFQELIPQDLINVFDEREL
AN1339.1  	LKPGGEDIPVTNENKHEYVE2LVTEWKIVKRVEEQFNAFMSGFNELIPADLVNVFDEREL
          	* *.*.:* ******:***: *:.:*:* **: :**.**  **:**** **:********

MG07255.1 	ELLIGGIAEIDVDDWKKHTDYRGYTEQDEVIQFFWQTVRSWDGEQKSRLLQFTTGTSRIP
NCU03947.1	ELLIGGIAEIDVDDWKKHTDYRGYTESDEVIQFFWQTVRSWDGEQKSRLLQFTTGTSRIP
FG10272.1 	ELLIGGIAEIDVDDWKKHTDYRGYTESDEVVQNFWATVRSWDGEQKSRLLQFTTGTSRIP
AN1339.1  	ELLIGGIADIDVDDWKKHTDYRGYQEQDEVIQNFWKIVRTWDAEQKSRLLQFTTGTSRIP
          	********:*************** *.***:* **  **:**.*****************

MG07255.1 	VNGFKDLQGSDGPRRFTIEKAGEINNLPKAHTC2FNRLDLPPYKSLEMLQQKMTIAVEET
NCU03947.1	VNGFKDLQGSDGPRRFTIEKAGEITNLPKAHTC2FNRLDLPPYKSLEMLQQKLTIAVEET
FG10272.1 	VNGFKDLQGSDGPRRFTIEKAGEITNLPKAHTC2FNRLDLPPYKSLEMLQQKLTIAVEET
AN1339.1  	VNGFKDLQGSDGPRRFTIEKSGDPIALPKSHTC2FNRLDLPPYKSHEVLEHKLSIAVEET
          	********************:*:   ***:*** *********** *:*::*::******

MG07255.1 	MGFGQE
NCU03947.1	MGFGQE
FG10272.1 	MGFGQE
AN1339.1  	LGFGQE
          	:*****
```
